# Exergetic Analysis and Design of a Mechanical Compression Stage—Application for a Cryogenic Air Separation Plant

**DOI:** 10.3390/e27050532

**Published:** 2025-05-16

**Authors:** Adalia Andreea Percembli (Chelmuș), Arthur Dupuy, Lavinia Grosu, Daniel Dima, Alexandru Dobrovicescu

**Affiliations:** 1 Department of Engineering Thermodynamics, National University of Science and Technology Politehnica București, 060042 Bucharest, Romania; adalia.chelmus@yahoo.com; 2 Laboratoire Energétique Mécanique Electromagnétisme (LEME), IUT de Ville d’Avray, 50 rue de Sèvres, 92410 Ville d’Avray, France; arthur.dupuy@parisnanterre.fr (A.D.); mgrosu@parisnanterre.fr (L.G.)

**Keywords:** exergy analysis, exergoeconomics, compressor, heat exchanger efficiency, cryogenics

## Abstract

This study focuses on the compression area of a cryogenic air separation unit (ASU). The mechanism of exergy consumption in the compressor was revealed. The influence of the compression ratio and of the isentropic efficiency per stage give arguments for proper choice of these decisional parameters. For the purchase cost of the compressor, an exergoeconomic correlation based on the exergetic product represented by the compression ratio and the isentropic efficiency as the Second Law coefficient of performance was used instead of the common thermo-economic one based only on the cost of materials. The impact of the suction temperature on the compressor operating performance is shown, making the gap between the compression stage and the associated intercooler. After optimization of the global system, a specific exergy destruction is assigned to each inter-stage compression cooler. To fit this optimum exergy consumption, a design procedure for the inter-stages and final coolers based on the number of heat transfer units (NTU-ε) method and the number of exergy units destroyed (NEUD) is shown. Graphs are provided that make the application of the method straightforward and much easier to use compared to the usual logarithmic mean temperature difference. A 25% increase in the compression ratio per stage leads to a decrease in the exergy efficiency of 3%, while the purchase cost of the compressor rises by 80%. An increase in the isentropic efficiency of the compressor from 0.7 to 0.85 leads to an increase in the exergetic performance coefficient of 21%, while the compressor purchase cost triples.

## 1. Introduction

Cryogenic Air Separation Units (ASUs) are indispensable in modern industry and technology, allowing efficient separation of atmospheric air into its primary components, oxygen, nitrogen, and argon, at extremely low temperatures. These gases are essential for a wide range of applications, from steel and chemical production to medical oxygen supply and food preservation. ASUs also play a crucial role in advancing green energy technologies, such as carbon capture and storage, gasification, and the integration of renewable energy by storing excess energy in liquid air [1,2]. As the only cost-effective method for large-scale air separation, optimizing the ASU is essential to align with current energy trends focused on efficiency, emission reduction, and sustainability. Their improvement remains a key area of academic and industrial research driven by the need to reduce energy consumption and strengthen their role in the transition to a low-carbon energy future.

The ASU uses the differences between the boiling temperatures of the air components to achieve separation through a process of compression, cooling, and distillation. First, atmospheric air is compressed, purified, and cooled at extremely low temperatures until it is partially liquefied. In the subsequent distillation columns, the liquefied air is separated based on the distinct boiling temperatures of its components. Nitrogen, being lighter, rises and is exhausted from the top of the column, whereas oxygen, being heavier, is collected at the bottom. Often, a secondary stream extracts argon from an intermediate zone.

The cryogenic air separation process, although effective in obtaining high-purity gases, is associated with significant energy consumption and irreversible thermodynamic losses; thus, numerous studies have been conducted to reduce this consumption.

Song et al. [3] studied the optimization of liquid detent turbines in ASUs using Joule-Thomson valves. By adjusting the rotor and cone geometry, they significantly reduced cavitation and swirl flow, thereby increasing the isentropic efficiency of the turbine. This study demonstrated that this approach improves ASUs performance and energy efficiency.

The importance of considering both energy quantity and quality in exergetic analysis, especially when evaluating mechanical energy inputs such as compression, is supported by Javed et al. [4] in their study on thermally integrated distillation columns. They emphasized that using mechanical energy, which is an ordered form of energy, to achieve an equivalent reduction in thermal energy (disordered form) is not always practical from an exergetic efficiency point of view. This principle is relevant when analyzing the exergetic efficiency of mechanical compression stages, where the quality of the energy introduced must be carefully weighed against the energy savings achieved.

In their study, Fernandes et al. [1] performed an exergetic analysis of a power plant integrated with an air separation unit. The study identified the main sources of exergy destruction within the ASU, highlighting the cryogenic heat exchanger and main air compressor as significant contributors. The researchers also concluded that locations with low altitude and high ambient pressure are preferred for ASU operations, as these lead to lower power requirements for the main air compressor.

The potential for energy savings in ASUs has also been explored by Cong et al. [5], who studied thermally integrated distillation technology and reported energy savings of more than 30% compared to conventional methods. Their system includes rectifying and stripping sections connected by a compressor and a throttling valve, with heat exchangers allowing thermal coupling. A fully thermally coupled structure transfers heat between the high- and low-pressure columns, approaching ideal configurations, thereby increasing the energy utilization and reducing the loads on the condenser and reboiler for improved efficiency.

In their study, Kotowicz et al. [6] analyzed four variants of supercritical power plants using oxygen as fuel and compared the air separation technologies and types of boilers used. This study showed that although cryogenic ASUs are a well-established technology, they have high auxiliary energy consumption, which reduces plant efficiency and increases costs. However, advanced membrane-based ASU technologies, particularly high-temperature membrane systems, significantly reduce energy consumption and increase efficiency. These results emphasize the importance of developing more efficient ASU technologies for various applications, as their performance has a major impact on energy systems in multiple industrial and power generation contexts.

Another approach to improve ASU efficiency is waste heat recovery using Organic Rankine Cycles (ORCs) [7]. This study explored the use of ORCs to recover heat from the compression zone of a cryogenic ASU by converting it into mechanical energy. The optimized ORC system reduced the total electricity consumption by 6% by generating a mechanical power of 1991 kW, demonstrating its potential to improve the performance of the ASU by harnessing waste heat.

The efficiency of the compression process is a key factor in improving the performance of air separation systems, with direct implications for both energy consumption and investment cost. The first stage of ASU operation is to compress atmospheric air and remove moisture, carbon dioxide, and other impurities, which, without proper control, can freeze and affect the operation of cryogenic equipment. An optimized compression ratio not only increases the efficiency of the cooling process but also facilitates more efficient liquefaction of the air, which is essential before it enters the distillation column. Given that the compression process accounts for a sizable fraction of the fuel-related exergy destruction of an ASU [8], any improvement at this stage has a significant impact on the overall operational cost and energy efficiency.

The highest electricity consumption in a cryogenic air separation plant occurs in the compression zone. Understanding the functional mechanisms offers the possibility of an optimal choice of operating parameters for the compression stage.

**Figure 1:**
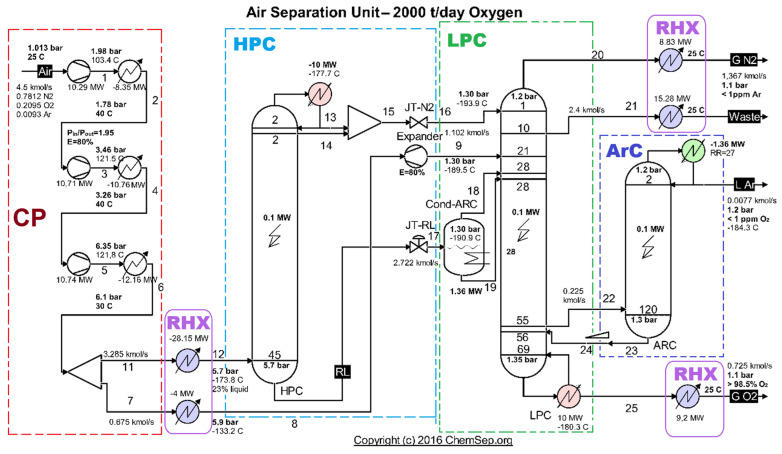
Schematic of the cryogenic air separation installation–functional areas. Source: Harry Kooijman (2006) chemsep.org [8,9].

The cryogenic air separation unit (ASU) (Figure 1) is composed, in order, of CP—compression area, HPC—high-pressure separation column, LPC—low-pressure separation column, and ArC—argon separation column.

The compression area of the ASU (CP) consists of three compression stages linked by two air intercoolers and an aftercooler (Figure 1).

Exergetic analysis specifies the location and magnitude of electricity consumption and the influence of decisional and performance parameters on the efficiency of the compression stages and the separation system as a whole.

The exergetic method combined with economic analysis can provide a viable strategy for optimizing a complex energy system based on the minimum total cost of the system product, the cost represented by the sum of the operating cost, and depreciation of the invested capital.

The optimal solution determined based on the exergoeconomic analysis is characterized by the local exergy consumption (electricity as fuel of the global system), which corresponds to a certain size and performance characteristic of the equipment that constitutes each functional area.

This study aims to establish a sizing method for compression zone air coolers for a specified exergy destruction following an optimization procedure.

For the compression stages, the application of the exergetic analysis method allows the unveiling of the links between the electrical energy consumption (exergy), to compensate for the useful energy dissipations caused by the internal irreversibilities, and the decisional functional parameters, such as the compression ratio on the stage, suction temperature, and compressor performance quality parameter (isentropic efficiency), which is related to its purchase cost.

Scientists have noticed the importance of the consequences of the second law of thermodynamics and the concept of exergy in analysis methods promoted [10,11,12,13,14]. Methods are sought to find the optimal functional and constructive regimes whose objective functions are based on the real performance criteria of the systems.

A method for rating a gas-to-gas heat exchanger based on the exergy concept and exergy effectiveness was developed by Creyx et al. [15]. The analysis shows how exergy destruction is shared among the key parts of the system.

Faris et al. [16] established a method of failure grouping and priority ranking in the case of operating a gas compression plant. The proposed method can be used in the management of combustible gas compression installations to decrease unit downtime time and increase plant efficiency and safety.

Souifi et al. [17] present a method for rating an air-to-air heat exchanger for an air conditioning system. The exchanger design was based on the NTU-ε method.

Zeitoun et al. [18] presented an energetic and exergetic analysis for an Earth—air heat exchanger accounting for the thermal energy gains of the air in contact with the Earth’s surroundings.

Li et al. [19] studied the performances and optimization of a series of heat exchangers based on exergoeconomic principles. The heat exchangers were equipped with an ORC supplied with heat recovered from a marine diesel engine.

Hu et al. [20] proposed the use of a pre-cooling air compressor to cool the air at the inlet to the compressor to save energy input.

Poljak et al. [21] studied the performance of a two-stage compressor on an LNG carrier from an energetic and exergetic perspective. They noticed a significant influence of environmental temperature. The influence of precooling between compression stages was also studied.

Sun et al. [22] used synergetics to optimize a multi-compressions system.

Zhang et al. [23] analyzed, based on the exergy concept, a compressed air engine with turbines, giving suggestions for reduction of exergy losses and increasing global efficiency.

Mehdizadeh-Fard et al. [24] proposed a simple approach for exergy analysis of a heat exchanger network. Their study indicated that the major exergy destruction source was the heat transfer across a finite temperature difference irreversibility.

Naeiji et al. [25], to compare the processes of cryogenic distillation and chemical scrubbing for biogas and hydrogen production, employed exergetic and economic analysis. In the case of cryogenic distillation, they note that the greatest exergy destruction occurs in the compressor. For the compressor, they obtained overall results provided by the Aspen HYSYS simulator, without delving into the details of the processes to identify the causes of irreversibility to offer solutions for mitigating the negative effects. In the economic analysis, equipment costs are specified, but they are not correlated with their performance or the output produced.

To compare different cryogenic cycles used for natural gas liquefaction, Atasbak et al. [26] used exergetic analysis. The study allowed them to highlight the values of stage compression ratios for which the installation’s coefficient of performance is maximized. The exergetic analysis enabled them to make decisions regarding structural modifications to the process schemes. However, aspects such as the performance of the compression process—expressed through isentropic efficiency—or the effect of suction temperature on compressor behavior were not addressed in their research, aspects which are analyzed in detail in the present study.

In their comparative analysis of various cryogenic cycles, Sijo K. and Rijo Jacob Thomas [27] consider exergetic analysis to be the most appropriate method for identifying optimal operational and design solutions. Their study concludes that irreversibility can be reduced more effectively through internal cooling via expansion in expansion machines rather than by using an external precooling system. The simulation is conducted using Aspen HYSYS, focusing on overall performance metrics for each system component, without investigating the underlying sources of process irreversibilities.

Dorosz et al. [28] use exergetic analysis to optimize and compare natural gas liquefaction systems aimed at cold energy recovery and its use in refrigeration or air-conditioning systems. Among the recovery options, particular attention is given to systems using direct expansion or the Organic Rankine Cycle. In the optimization procedure, the study mentions the possibility of replacing shell-and-tube vaporizers with plate heat exchangers, but without providing detailed design considerations. The simulation is carried out using Aspen HYSYS V10 (36.0.0.249) software.

Dr. R.S. Mishra and Devendra Kumar [29] emphasize in their literature review the usefulness of exergetic analysis in the evaluation of cryogenic gas liquefaction systems. However, they do not reference any sources that provide equipment sizing methodologies based on a recommended level of exergy destruction.

To identify new refrigerants with superior properties for refrigeration systems operating at cryogenic temperatures, Qin et al. [30] use exergetic efficiency to evaluate system performance. Their analysis focuses on the compressor and the throttle valve, which are characterized by the highest exergy consumption within the system.

Bibliographic research highlights the interest in increasing the performance of the compression stage of cryogenic air separation installations but also the lack of an extensive bibliography in this field. It appears evident that there is a need to build a calculation method to optimize the compression stage of the ASU based on the thermodynamics of irreversible processes, the only one that can approach reality by considering both the first and second laws.

In the cited works, the exergy optimization procedure was performed for a device or functional area of a system, taken as a whole, without entering the intimacy of the functional processes and without highlighting the genesis of irreversible processes. In the present work, the mechanism of exergy consumption (destruction) will be pointed out, highlighting the share of each functional and constructive parameter in the exergy consumption that supplies each component part of the system. In addition, in the present work, a dimensional calculation methodology of the heat transfer surface for a specified exergy destruction is also offered. The exergy destruction distributed to each functional area results from an exergy optimization procedure of the entire system.

## 2. The Method of Analysis and Design of the ASUs Compression Zone

Bucsa et al. [8] offer a detailed analysis of the processes in a cryogenic air separation unit.

The compression zone is characterized by the largest share of exergy destruction in the overall system fuel (Table 1, Table 2 and Table 3).

Table 1, Table 2 and Table 3 present in order the electrical power consumption for each compression stage (Table 1), the exergy destructions in each stage (Table 2), and the exergy losses with heat discharged in the intermediate and final coolers, to which are added the exergy destructions attached to the friction process when the compressed gas passes through the heat exchangers (Table 3).

The sum of exergy losses and destruction in the compression zone is

(1) $${\dot{I}}_{Z,cp}={\dot{I}}_{cp}+{\dot{L}}_{Q}=4.958+6.781=11.793\;MW.$$

The share of exergy losses and destruction in the compression zone in the fuel consumption of the global installation is

(2) $$\psi_{cp}=\frac{{\dot{I}}_{Z,cp}}{\left|{\dot{W}}_{1}\right|+\left|{\dot{W}}_{2}\right|+\left|{\dot{W}}_{3}\right|}=\frac{11.793}{31.755}=0.37.$$

This exergy destruction characterizes both the compression process and the heat transfer to the ambient environment in the coolers.

To study the mechanism of exergy destruction in the compression zone and its influence on the zone performance, the following key parts were analyzed in the following order:

(a) The compression stage.
(b) The intermediary and final coolers.

### 2.1. Exergetic Analysis of the Compression Stage

Compressors have the role of achieving an increase in the pressure of the air subjected to the separation process.

The compression process, as the main process of the compressor, is generally accompanied not only by an increase in pressure but also by an increase in temperature.

In the case of an air compressor operating at temperatures above ambient temperature, an increase in temperature during the compression process has an undesirable effect.

**Figure 2:**
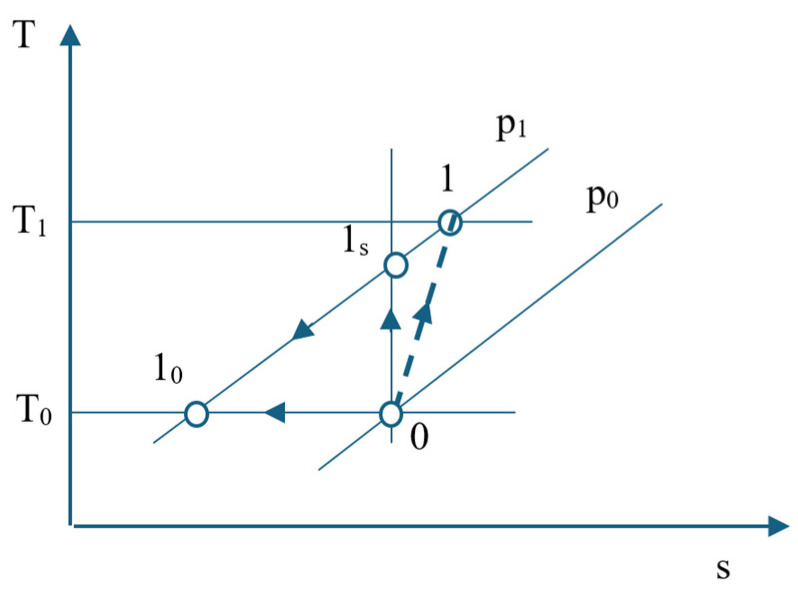
T-s diagram representation of the first stage compression process.

It is considered a compression stage, and neglecting the effect of pressure losses in suction and discharge, the exergy destruction when compressing one kmol of air will be followed. In Figure 2, the compression process in the first stage of the ASU compressor is represented in a T-s diagram.

The process is considered irreversible adiabatic. The entropy generation follows the irreversible adiabatic compression.

The exergy balance for a control volume is as follows [31]

(3) $$\sum {Ex}_{q}=\sum {Ex}_{o}-\sum {Ex}_{i}+\sum W+\sum I,$$

where ${Ex}_{q}$ and W respectively represent the exergy of heat and mechanical work exchanged by the control volume with its surroundings, ${Ex}_{o}$ and ${Ex}_{i}$ are exergies of outlet and inlet currents, and I is an exergy consumption or destruction.

In the exergy balance equation, the exergy product of the system and the corresponding fuel that is consumed to obtain the product should be revealed to determine the exergetic coefficient of performance. In terms of fuel, product, loss, and destruction, the exergy balance becomes as follows:

(4) F=P+L+I.

Accounting for Equation (4), exergetic COP is written as follows:

(5) $${COP}_{ex}=\frac{P}{F}=1-\frac{L+I}{F}\;.$$

An exergy destruction could be determined based on the Gouy-Stodola theorem:

(6) $$I=T_{0}\cdot S_{gen}.$$

The flow exergy of a current entering or leaving the system could be calculated as

(7) $$Ex=H-H_{0}-T_{0}\cdot \left(S-S_{0}\right),$$

where subscript “0” represent the system in thermo-mechanical equilibrium with its environment.

In the case of the compression process, the exergetic balance written for 1 kmol of air becomes as follows:

(8) $$\sum {\bar{ex}}_{q}=∆\bar{ex}+{\bar{w}}_{cp}+\sum \bar{i},$$

where

(9) $$\sum {\bar{ex}}_{q}=0\left(adiabatic\;compression\right),$$

(10) $${\bar{w}}_{cp}<0,{\bar{w}}_{cp}=-\left|{\bar{w}}_{cp}\right|,$$

(11) $$∆\bar{ex}={\bar{ex}}_{1}-{\bar{ex}}_{0}={\bar{h}}_{1}-{\bar{h}}_{0}-T_{0}\left({\bar{s}}_{1}-{\bar{s}}_{0}\right).$$

Air at the pressures and temperatures characteristic of the compression process behaves as a perfect gas, as a result:

(12) $$∆\bar{ex}={\bar{c}}_{p}\left(T_{1}-T_{0}\right)-T_{0}\left({\bar{c}}_{p}ln\frac{T_{1}}{T_{0}}-\bar{R}ln\frac{p_{1}}{p_{0}}\right).$$

From the calculation equation of the exergy variation (12), the part related to the temperature variation (thermal exergy variation) is separated from the part given by the pressure variation (mechanical exergy variation):

(13) $$∆\bar{ex}=∆{\bar{ex}}^{T}+∆{\bar{ex}}^{M}=\left[{\bar{c}}_{p}\left(T_{1}-T_{0}\right)-T_{0}\cdot {\bar{c}}_{p}ln\frac{T_{1}}{T_{0}}\right]+T_{0}\cdot \bar{R}\cdot ln\frac{p_{1}}{p_{0}},\;$$

where

(14) $$∆{\bar{ex}}^{T}={\bar{c}}_{p}\left(T_{1}-T_{0}\right)-T_{0}\cdot {\bar{c}}_{p}ln\frac{T_{1}}{T_{0}}=\bar{q}-T_{0}\frac{\bar{q}}{T_{1-1_{0}}}=\bar{q}\left(1-\frac{T_{0}}{T_{1-1_{0}}}\right),$$

represents the exergy of a heat given up in an isobaric cooling process from the temperature $t_{1}$ to the temperature $t_{0}$ of the ambient medium.

In relation (14), $T_{1-1_{0}}$ is the average thermodynamic temperature of the isobaric cooling process $1-1_{0}$ as follows:

(15) $$T_{1-1_{0}}=\frac{\bar{q}}{∆{\bar{s}}_{1-1_{0}}}=\frac{{\bar{c}}_{p}\left(T_{1}-T_{0}\right)}{{\bar{c}}_{p}ln\frac{T_{1}}{T_{1_{0}}}}=\frac{T_{1}-T_{0}}{ln\frac{T_{1}}{T_{1_{0}}}}\;.$$

$∆{\bar{ex}}^{T}$ represents an exergy loss or, if the boundary of the system passes through the environment, it falls into the category of exergy destruction $\left({\bar{i}}_{∆T}\right)$.

Continuing the analysis of the exergy variation expression, it is observed that

(16) $$∆{\bar{ex}}^{M}=T_{0}\cdot \bar{R}\cdot ln\frac{p_{1}}{p_{0}}=\left|{\bar{w}}_{{cp}_{T0}}\right|$$

is the absolute value of the isothermal compression mechanical work at the ambient temperature $T_{0}$.

$∆{\bar{ex}}^{M}$ represents the minimum mechanical work of compression from $p_{0}$ to $p_{1}$. In this process, the system only exchanges heat with the environment under reversible conditions (at $T_{0}$).

The irreversibility of the compression process is calculated using the Gouy-Stodola theorem [32]:

(17) $${\bar{i}}_{cp}=T_{0}\left({\bar{s}}_{1}-{\bar{s}}_{0}\right).$$

Considering expressions (9)–(17), the exergetic balance Equation (8) becomes as follows:

(18) $$\left|{\bar{w}}_{cp}\right|=\left|{\bar{w}}_{{cp}_{T0}}\right|+{\bar{i}}_{∆T}+{\bar{i}}_{cp}.$$

$\left|{\bar{w}}_{{cp}_{T0}}\right|$ is the isothermal compression mechanical work at the ambient temperature ($T_{0}$) representing the desired product of the compression process, while $\left|{\bar{w}}_{cp}\right|$ represents the actual fuel.

According to Equation (5), the exergetic performance coefficient of compression is as follows:

(19) $${COP}_{ex}^{cp}=\frac{\left|{\bar{w}}_{{cp}_{T0}}\right|}{\left|{\bar{w}}_{cp}\right|}=1-\frac{{\bar{i}}_{∆T}+{\bar{i}}_{cp}}{\left|{\bar{w}}_{cp}\right|}\;.$$

It is noted that

(20) $$\left|{\bar{w}}_{cp}\right|={\bar{h}}_{1}\left(T_{1}\right)-{\bar{h}}_{0}\left(T_{0}\right)\;,$$

(21) $${ɳ}_{s,cp}=\frac{h_{1s}-h_{0}}{h_{1}-h_{0}}\;,$$

(22) $$h_{1}\left(T_{1},p_{1}\right)=h_{0}\left(T_{0},p_{0}\right)+\frac{h_{1s}\left(T_{1s},p_{1}\right)-h_{0}\left(T_{0},p_{0}\right)}{{ɳ}_{s,cp}}\;,$$

and

(23) $$T_{1s}=T_{0}{\left(\frac{p_{1}}{p_{0}}\right)}^{\frac{k-1}{k}}=T_{0}\cdot \pi^{\frac{k-1}{k}},$$

where $\pi =\frac{p_{1}}{p_{0}}$ is the pressure increase ratio on the compression stage.

It follows that

(24) $${COP}_{ex}^{cp}=\frac{T_{0}\cdot \bar{R}\cdot ln\pi }{{\bar{h}}_{1}\left(T_{1}\right)-{\bar{h}}_{0}\left(T_{0}\right)}=\frac{T_{0}\cdot \bar{R}\cdot ln\pi }{{\bar{h}}_{1}\left(T_{0,}\pi ,{ɳ}_{s,cp}\right)-{\bar{h}}_{0}\left(T_{0}\right)}\;.$$

**Figure 3:**
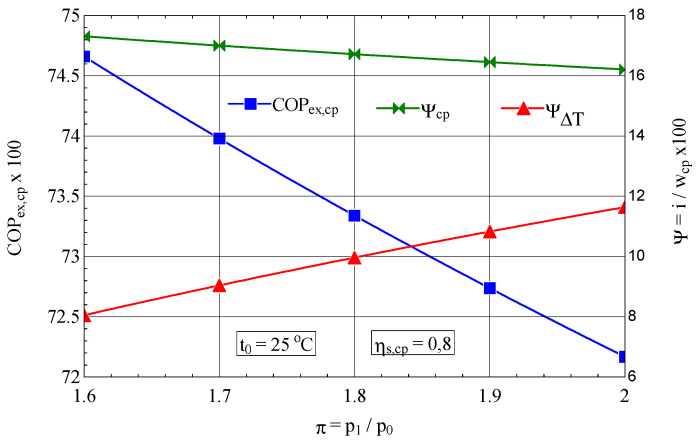
The exergetic performance coefficient of the compression stage and the share of exergy destruction from the mechanical power input as a function of the compression ratio.

It is interesting to observe the variation of the exergetic performance coefficient of the compressor and the weights of the exergy destructions with the variation of the compression ratio and the isentropic compression efficiency (Figure 3).

The weights of exergy destruction in the consumption of mechanical compression work are calculated as follows:

(25) $$\psi_{j}=\frac{{\bar{i}}_{j}}{\left|{\bar{w}}_{cp}\right|}\;.$$

In the case of increasing the compression ratio per stage, the exergetic performance coefficient decreases due to the unwanted increase in the share of the loss associated with the increase in the temperature of the discharged air ($\psi_{∆T}$), which is more pronounced than the decrease in the share of exergy destruction caused by internal frictions ($\psi_{cp}$) (Figure 3).

The 25% increase in the compression ratio π per stage leads to an increase in the share of exergy destruction due to the increase in gas temperature of $\psi_{∆T}=$ 43%, which, weighted by the decrease in exergy destruction due to friction of $\psi_{∆T}=$ 9%, leads to a decrease in exergy efficiency of 3%.

**Figure 4:**
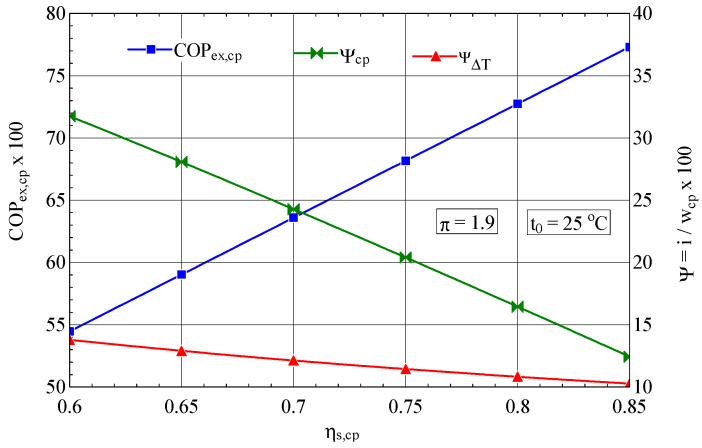
The exergetic performance coefficient of the compression stage and the share of exergy destruction from the mechanical power input as a function of the isentropic efficiency of the compression process.

The isentropic efficiency of compression has a major influence on the performance of the compression process; an increase in $\psi_{s,cp}$ from 0.7 to 0.85 leads to an increase in the exergetic performance coefficient of 21% (Figure 4).

As expected, the increase in the isentropic efficiency of the compressor leads to an increase in its exergetic performance coefficient and finally to a decrease in the consumption of electricity, the fuel of the overall system.

**Figure 5:**
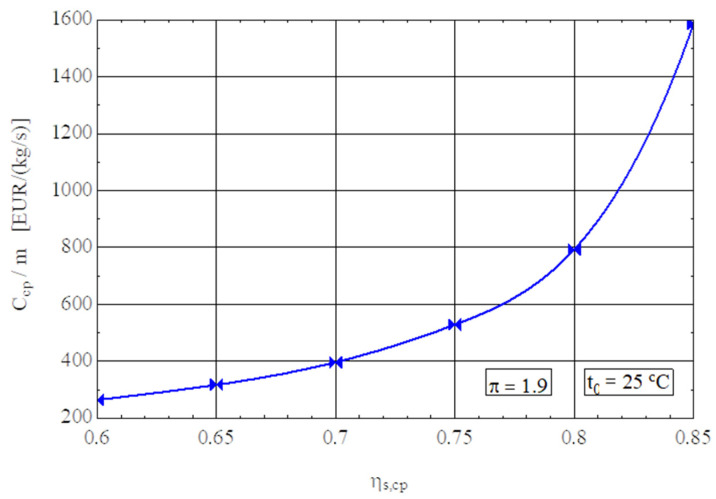
Purchase cost of the compressor in relation to 1 kg/s gas flow rate as a function of the isentropic efficiency ${ɳ}_{s,cp}$ of the compression stage.

The decrease in operating costs is achieved in this case by purchasing more expensive compressors, i.e., by increasing the amortization rate of capital investment (Figure 5).

The purchase cost of the compressor for 1 kg/s of compressed gas was calculated with the exergoeconomic correlation (26) [33] where the quality factor of the product (compressed gas) is the pressure increase ratio π and that of the compressor is the isentropic efficiency ${ɳ}_{s,cp}$.

(26) $$\frac{C_{cp}}{\dot{m}}=65.058\frac{\pi \cdot ln\pi }{0.9-{ɳ}_{s,cp}}\frac{EUR}{\frac{kg}{s}}$$

**Figure 6:**
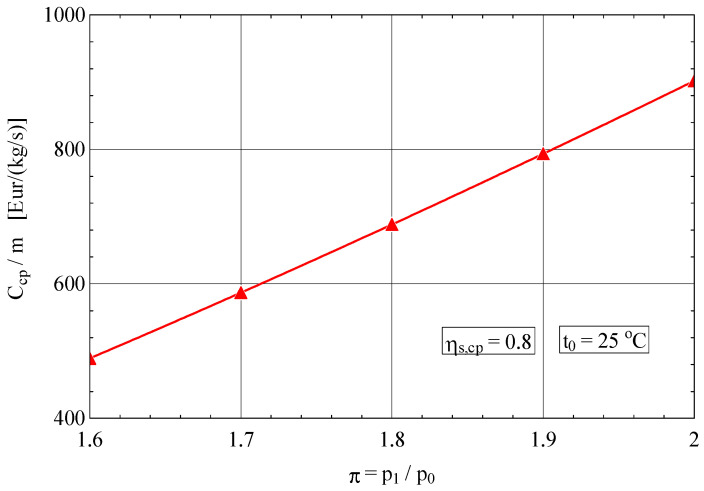
Purchase cost of the compressor in relation to 1 kg/s flow rate of compressed gas as a function of the compression ratio π.

As expected, the purchase cost of the compressor $\frac{C_{cp}}{m}$ for 1 kg/s compressed gas increases rapidly with these two quality indicators (Figure 5 and Figure 6).

In Equation (26), the maximum isentropic efficiency was taken ${ɳ}_{s,cp}=0.9$, for which the purchase cost of the compressor tends to infinite.

For an increase in the ${ɳ}_{s,cp}$ from 0.7 to 0.85, the compressor purchase cost triples (Figure 5).

For an increase in the compression ratio of 25%, the purchase cost of the compressor rises by 80%.

**Figure 7:**
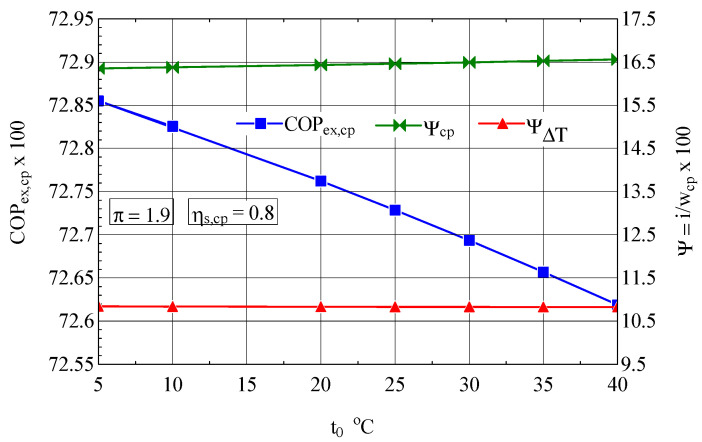
The exergetic performance coefficient of the compression stage and the share of exergy destruction from the mechanical power input as a function of the ambient temperature (suction temperature for the first compression stage (Figure 1).

The suction temperature also plays an important role in the economy of the compression stage, which is highlighted by the exergetic analysis method (Figure 7). When the suction temperature increased, the exergetic performance coefficient ${COP}_{ex,cp}$ of the compression stage decreased.

The decrease in the suction temperature at the compression stage inlet is the part played by the coolers.

### 2.2. Design of Intercoolers and Final Cooler Based on Optimal Entropy Generation (Optimal Exergy Destruction)

The intercooler or aftercooler of a multi-stage compressor is characterized by heat transfer to the ambient environment and pressure losses due to friction.

The heat discharged into the environment has a potential use for which resources have been consumed somewhere in the system, and resources are also consumed through friction.

Although the evacuation of heat is a thermal process and the overcoming of frictional forces is a mechanical process, they are both part of the category of irreversible processes and have the effect of consuming (or destroying) exergy (useful energy [34]).

Consequently, the two cumulative effects (of heat transfer and pressure loss) can be evaluated using a single indicator, namely, the exergy destruction caused by the irreversibility of the processes.

The exergy destroyed in a heat exchanger can be calculated based on the Gouy-Stodola theorem (Equation (6)).

#### 2.2.1. Thermal Efficiency of a Heat Exchanger

**Figure 8:**
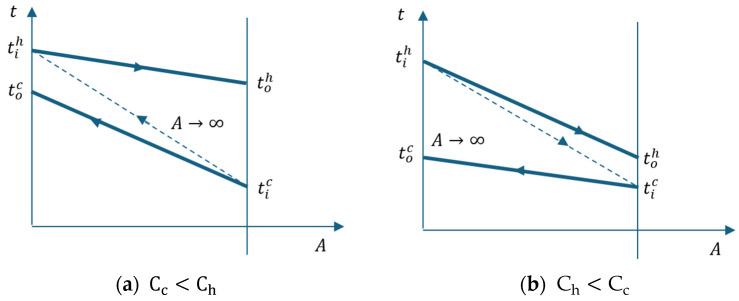
Heat exchanger temperature variation with the surface (**a**) $C_{c}<C_{h}$; (**b**) $C_{h}<C_{c}$.

Figure 8 shows the temperature-heat exchange surface diagram of the cooler.

The thermal efficiency of a heat exchanger is calculated as the ratio of the heat exchanged in the discussed case to the maximum heat ${\dot{Q}}_{max}$ that can be exchanged in an apparatus with an infinite surface area.

(27) $$\varepsilon =\frac{\dot{Q}}{{\dot{Q}}_{max}}=\frac{C_{h}\left(t_{i}^{h}-t_{o}^{h}\right)}{C_{min}{(t}_{i}^{h}-t_{i}^{c})}=\frac{C_{c}\left(t_{o}^{c}-t_{i}^{c}\right)}{{C_{min}(t}_{i}^{h}-t_{i}^{c})}$$

where $C_{min}$ is the lower calorific capacity of the two currents.

With respect to the energy balance, the highest temperature variation was associated with the lowest calorific capacity.

Considering that the cooling fluid is water, in the case of the intermediate or final cooler of the compressor, the lowest calorific capacity is on the air side (hot stream) ($C_{min}=C_{h}$).

It follows that

(28) $$\varepsilon =\frac{\left(t_{i}^{h}-t_{o}^{h}\right)}{{(t}_{i}^{h}-t_{i}^{c})}$$

#### 2.2.2. Number of Heat Transfer Units NTU

The definition of the number of heat transfer units (NTU) is as follows [35]:

(29) $$NTU=\frac{U\cdot A}{C_{min}}=\frac{1}{C_{min}}\int_{0}^{A}U\left(A\right)dA$$

In the case of the analyzed cooler $C_{min}=C_{h}$ and from Equation (28), it follows that

(30) $$\varepsilon =\frac{\left(t_{i}^{h}-t_{o}^{h}\right)}{∆t_{0}}$$

where $∆t_{0}=t_{i}^{h}-t_{i}^{c}$.

From the last equality of relation (27), it follows that

(31) $$\frac{\left(t_{i}^{h}-t_{o}^{h}\right)}{∆t_{0}}=\frac{C_{c}}{C_{h}}\cdot \frac{t_{o}^{c}-t_{i}^{c}}{∆t_{0}}=\frac{C_{max}}{C_{min}}\cdot \frac{t_{o}^{c}-t_{i}^{c}}{∆t_{0}}$$

Identifying relations (30) and (31), we obtain the following:

(32) $$\varepsilon =\frac{\left(t_{i}^{h}-t_{o}^{h}\right)}{∆t_{0}}=\frac{C_{max}}{C_{min}}\cdot \frac{t_{o}^{c}-t_{i}^{c}}{∆t_{0}}$$

**Figure 9:**
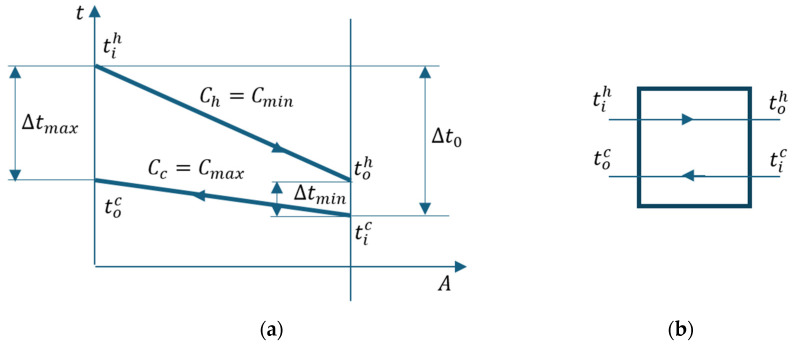
Cooler. (**a**) t-A diagram; (**b**) flow chart.

Figure 9 marks the temperature differences between the two streams at the ends of the heat exchanger and the temperature difference $∆t_{0}$ between the two streams at the entrance to the device.

Following the t-A diagram in Figure 9, the following relationships are deduced:

(33) $$∆t_{min}=∆t_{0}-\left(t_{i}^{h}-t_{o}^{h}\right)=∆t_{0}-\varepsilon \cdot ∆t_{0}=\left(1-\varepsilon \right)∆t_{0}$$

(34) $$∆t_{max}=t_{i}^{h}-t_{o}^{c}=\left(t_{i}^{h}-t_{o}^{h}\right)-\left(t_{o}^{c}-t_{i}^{c}\right)+\left(t_{o}^{h}-t_{i}^{c}\right)$$

where from the relation (32),

(35) $$t_{i}^{h}-t_{o}^{h}=\varepsilon \cdot ∆t_{0}$$

(36) $$t_{o}^{c}-t_{i}^{c}=\varepsilon \cdot ∆t_{0}\frac{C_{min}}{C_{max}}$$

and from (33),

(37) $$t_{o}^{h}-t_{i}^{c}=∆t_{min}=\left(1-\varepsilon \right)∆t_{0}$$

Considering Equations (35)– (37), relation (34) becomes as follows:

(38) $$\begin{array}{l} ∆t_{max}=t_{i}^{h}-t_{o}^{c}=\varepsilon \cdot ∆t_{0}-\varepsilon \cdot ∆t_{0}\frac{C_{min}}{C_{max}}+\left(1-\varepsilon \right)∆t_{0}=∆t_{0}\left(\varepsilon -\varepsilon \frac{C_{min}}{C_{max}}+1-\varepsilon \right)\\ \;\;\;\;\;\;=\left(1-\varepsilon \frac{C_{min}}{C_{max}}\right)∆t_{0} \end{array}$$

Knowing the temperature differences at the ends of the heat exchanger, the average logarithmic temperature difference between the two currents can be calculated:

(39) $$∆t_{m}=\frac{∆t_{max}-∆t_{min}}{ln\frac{∆t_{max}}{∆t_{min}}}=\frac{∆t_{0}\cdot \varepsilon \left(1-\frac{C_{min}}{C_{max}}\right)}{ln\frac{1-\varepsilon \frac{C_{min}}{C_{max}}}{1-\epsilon }}$$

Considering that $C_{min}=C_{h}$, it follows that

(40) $$NTU=\frac{U\cdot A}{C_{h}}$$

and

(41) $$\dot{Q}=C_{h}\left(t_{i}^{h}-t_{o}^{h}\right)=U\cdot A\cdot ∆t_{m}$$

The number of heat transfer units becomes

(42) $$NTU=\frac{U\cdot A}{C_{h}}=\frac{t_{i}^{h}-t_{o}^{h}}{∆t_{m}}$$

Substituting relation (42), the expressions of the temperature difference on the hot stream (Equation (35)) and the average temperature difference (Equation (39)), we obtain the following:

(43) $$NTU=\frac{\epsilon \cdot ∆t_{0}}{\frac{∆t_{0}\cdot \epsilon \left(1-\frac{C_{min}}{C_{max}}\right)}{ln\frac{1-\varepsilon \frac{C_{min}}{C_{max}}}{1-\varepsilon }}}=\frac{ln\frac{1-\varepsilon \frac{C_{min}}{C_{max}}}{1-\varepsilon }}{1-\frac{C_{min}}{C_{max}}}$$

The relation (43) can be rewritten as follows:

(44) $$\left(1-\frac{C_{min}}{C_{max}}\right)NTU=ln\frac{1-\varepsilon \frac{C_{min}}{C_{max}}}{1-\varepsilon }$$

or

(45) $$e^{NTU\left(1-\frac{C_{min}}{C_{max}}\right)}=\frac{1-\varepsilon \frac{C_{min}}{C_{max}}}{1-\varepsilon }$$

Equation (45) can be solved in relation to the efficiency ε:

(46) $$\varepsilon =\frac{exp\left[NTU\left(1-\frac{C_{min}}{C_{max}}\right)\right]-1}{exp\left[NTU\left(1-\frac{C_{min}}{C_{max}}\right)\right]-\frac{C_{min}}{C_{max}}}$$

### 2.3. Exergy Destruction in the Cooler

The exergy destruction related to heat transfer across a finite temperature difference between the hot (T_h_) and cold bodies (T_c_) is as follows:

(47) $$I_{∆T}={Ex}_{Q}^{T_{h}}-{Ex}_{Q}^{T_{c}}=Q\left(1-\frac{T_{0}}{T_{h}}\right)-Q\left(1-\frac{T_{0}}{T_{c}}\right)=Q\left(\frac{T_{0}}{T_{c}}-\frac{T_{0}}{T_{h}}\right)=Q\cdot T_{0}\frac{T_{h}-T_{c}}{T_{c}\cdot T_{h}}$$

If we consider only the exergy destruction caused by the heat transfer to the cooling medium (water and finally the air from the cooling tower), considered with a good approximation to be the ambience medium, its expression is as follows:

(48) $${\dot{I}}_{∆T}=\dot{Q}\cdot T_{0}\frac{T_{h}-T_{c}}{T_{h}\cdot T_{c}}=\dot{Q}\left(1-\frac{T_{c}}{T_{h}}\right)$$

where $T_{0}≅T_{c}$.

Relating relation (48) to $\dot{Q}$ yields a dimensionless number that we call NEUD—the number of exergy units destroyed [36]:

(49) $$NEUD=\frac{{\dot{I}}_{∆T}}{\dot{Q}}=\left(1-\frac{T_{c}}{T_{h}}\right),$$

where

(50) $$T_{h}=\frac{T_{i}^{h}-T_{o}^{h}}{ln\frac{T_{i}^{h}}{T_{o}^{h}}}\;and\;T_{c}=\frac{T_{o}^{c}-T_{i}^{c}}{ln\frac{T_{o}^{c}}{T_{i}^{c}}}.$$

The outlet temperatures of the currents ($T_{o}^{h}$ and $T_{o}^{c}$) function of the inlet temperatures ($T_{i}^{h}$ and $T_{i}^{c}$) and the thermal efficiency (ε) were calculated from Equations (28) and (32) written with temperatures in [K].

The energy balance equation of the control volume of the heat exchanger (Figure 9) is as follows:

(51) $$C_{min}\left(T_{o}^{h}-T_{i}^{h}\right)+C_{max}\left(T_{o}^{c}-T_{i}^{c}\right)=0$$

Equation (28) gives the following:

(52) $$T_{o}^{h}=T_{i}^{h}-\varepsilon \left(T_{i}^{h}-T_{i}^{c}\right).$$

From Equation (32), we get the following:

(53) $$T_{o}^{c}=T_{i}^{c}+\frac{C_{min}}{C_{max}}\varepsilon \left(T_{i}^{h}-T_{i}^{c}\right).$$

### 2.4. Sizing the First Intercooler of the ASU Based on the NEUD

The functional characteristics of the compression zone coolers are shown in Table 4.

The first intermediate cooler of the compression zone is characterized by the ratio of the calorific capacities of the two currents

(54) $$C_{r}=\frac{C_{min}}{C_{max}}=\frac{{\dot{C}}_{h}}{{\dot{C}}_{c}}=0.14$$

**Figure 10:**
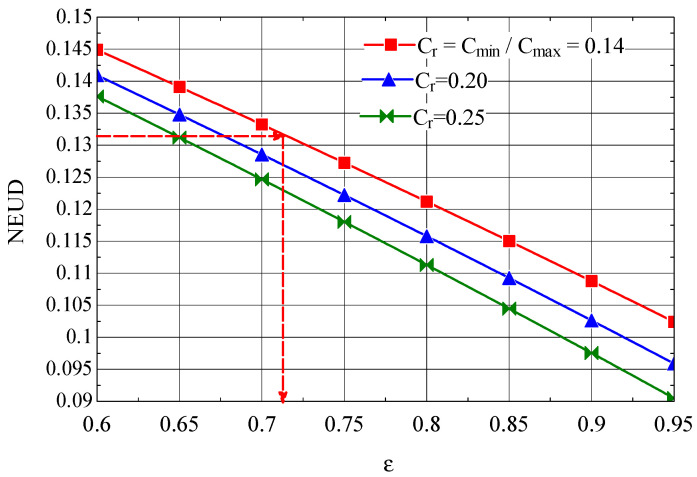
The number of destroyed exergy units NEUD as a function of the efficiency ε of the first intermediate cooler.

The NEUD—ε diagram built based on relations ((49), (50), (52), and (53)) is presented in Figure 10.

The average thermodynamic temperatures of the air (hot stream) in the first cooler (subscript 1) and of the cooling water (Equation (50)) are as follows:

$T_{h_{1}}=343.7\;K$ and $T_{c_{1}}=298.33\;K$, to which it corresponds, for the first intermediate cooler (Figure 1), ${NEUD}_{1}=0.132$ (Equation (49)).

From Figure 10, for $C_{r}=0.14,$ the ${NEUD}_{1}=0.132$ corresponds to a thermal efficiency $\varepsilon_{1}=0.71$.

Knowing the thermal efficiency of the heat exchanger for a specified number of destroyed exergy units, the number of heat transfer units NTU_1_ can be determined.

**Figure 11:**
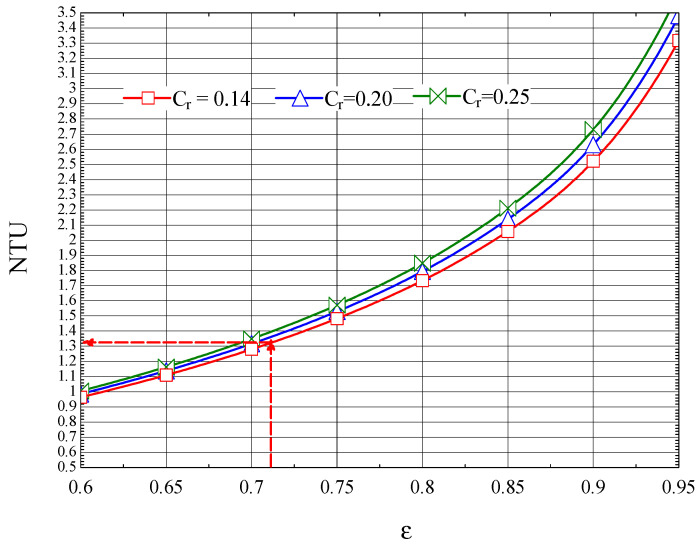
The number of NTU thermal units as a function of the efficiency ε of the first intercooler.

The NTU-ε plot for the first compression zone cooler, built up based on Equation (46), is shown in Figure 11.

The thermal efficiency $\varepsilon_{1}=0.71$ corresponds to ${NTU}_{1}=1.31$ (Figure 11).

According to the definition of the number of thermal units (Equation (29)), the following is obtained:

(55) $${\left(U\cdot A\right)}_{1}={\left(C_{min}\cdot NTU\right)}_{1}={\dot{C}}_{h}\cdot {NTU}_{1}=131.7\cdot 1.31=172.5\;\frac{kW}{K}$$

For an overall heat transfer coefficient $U_{1}=0.250\;\frac{kW}{m^{2}K}$, the heat transfer area becomes as follows:

(56) $$A_{1}={\left(\frac{C_{h}\cdot NTU}{U}\right)}_{1}=\frac{172.5}{0.250}=690\;m^{2}$$

## 3. Numerical Application for the Main Heat Exchanger of an ASU

The method was applied to the published case of the design of a Giauque–Hampson recuperative heat exchanger that equips an air liquefaction plant, presented by Barron R.F [37]. The results are identical to the ones presented in the published work.

The functional characteristics of the heat exchanger are specified in Table 5.

Unlike the previous case of ASU compression stage coolers, where $C_{min}=C_{h}$ (the calorific capacity of the water-cooled air), in the case of the Giauque–Hampson heat exchanger, $C_{min}=C_{c}$ represents the calorific capacity of the cold air from the low-pressure secondary stream.

The final calculation expression of NTU(ε), relations (43)–(46), remains the same regardless of which of the two currents that exchange heat between them has $C_{min}$.

Instead, the calculation expressions of the output temperatures of each current are modified because in the case of the recuperative exchanger $C_{min}=C_{c}$, and, consequently,

(57) $$\varepsilon =\frac{T_{o}^{c}-T_{i}^{c}}{T_{i}^{h}-T_{i}^{c}}$$

The system of Equations (51) and (57) leads to the following:

(58) $$T_{o}^{h}=T_{i}^{h}-\varepsilon \cdot C_{r}\left(T_{i}^{h}-T_{i}^{c}\right)$$

(59) $$T_{o}^{c}=T_{i}^{c}+\varepsilon \left(T_{i}^{h}-T_{i}^{c}\right)$$

The number of exergy units destroyed owing to the temperature difference between the fluids that exchange heat becomes as follows:

(60) $$NEUD=T_{0}\frac{T_{h}-T_{c}}{T_{h}\cdot T_{c}}$$

From the data specified in [37], it appears that the heat exchanger operates with NEUD = 0.426.

**Figure 12:**
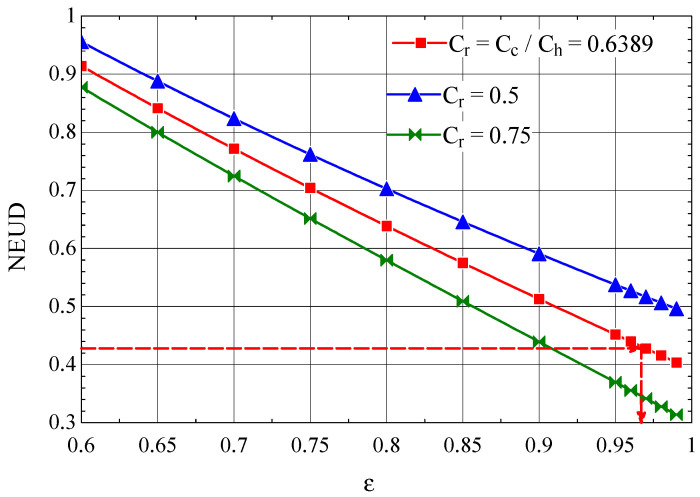
The number NEUD of destroyed exergy units depending on the efficiency of the Giauque–Hampson type recuperative heat exchanger [37].

For the Giauque–Hampson heat exchanger, the NEUD-ε diagram, built based on relations ((50), (58)–(60)), is presented in Figure 12.

**Figure 13:**
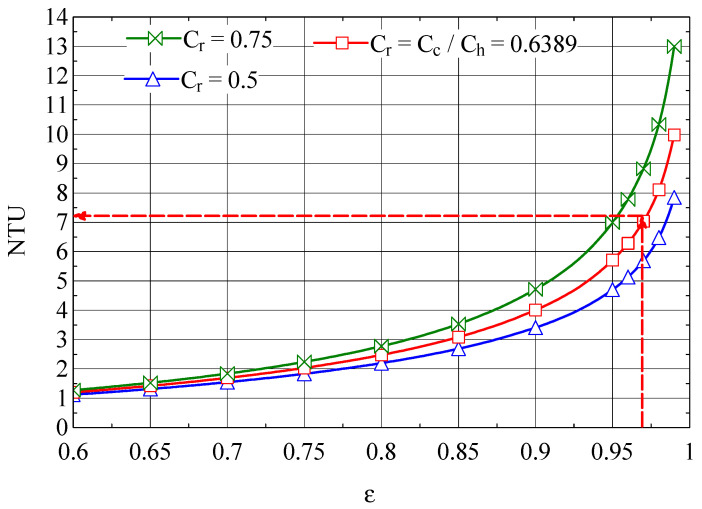
NTU-ε graph of the Giauque–Hampson recuperative heat exchanger [37].

The NTU-ε graph of the analyzed heat exchanger is shown in Figure 13.

From the graph in Figure 12, NEUD = 0.426 corresponds to ε = 0.9714, the efficiency for which from Figure 13 we find NTU = 7.16.

The overall heat transfer coefficient of the heat exchanger, $U_{o}=0.1959\;kW/\left(m^{2}K\right)$ [37], was calculated with reference to the smooth outer surface of the pipes.

From the definition expression of NTU,

(61) $$NTU=\frac{U_{0}\cdot A_{0}}{C_{c}}$$

the following results:

(62) $$A_{0}=\frac{NTU\cdot C_{c}}{U_{0}}=\frac{7.16\cdot 2.358}{0.1959}=86.18\;m^{2}$$

The heat transfer surface calculated by Baron [37] is identical to that calculated based on the NEUD(ε) and NTU(ε) numbers, the method proposed in the present paper.

## 4. Conclusions

This paper presents a method for estimating the impact of the operating and performance characteristics of the compression area on the input power based on the exergy concept.

For the compression stages, the influence of the compression ratio, suction temperature, and isentropic efficiency on the power input are shown.

After optimizing the compression area based on the optimum exergy destruction in each piece of equipment, the compression zone intercoolers and final cooler are designed based on the correlation given by the number of exergy units destroyed NEUD(ε) and the number of thermal units NTU(ε).

The method based on the numbers NEUD(ε) and NTU(ε) allows the quick specification of the value of the heat exchange surface and therefore of the investment cost of the heat exchanger for different values of the exergy destruction associated with the heat transfer, for specified values of the temperatures and heat capacities of the currents at the input of the heat exchanger.

The paper makes an important contribution to the understanding of the thermodynamics associated with the compression process. The splitting of the exergy of the flowing gas into its mechanical and thermal components provides a precise picture of how the exergy is consumed (destroyed) in the compression process, providing solutions for choosing optimal values of the decision-making parameters.

Correlating the choice of optimal operational parameters with the investment cost provides a precise picture of the balance between reducing exergy destruction (reducing operational cost) and increasing the cost of the invested capital.

A 25% increase in the compression ratio π per stage leads to an increase in the share of exergy destruction due to the increase in gas temperature of $\psi_{∆T}=$ 43%, which, weighted by the decrease in exergy destruction due to friction of $\psi_{cp}=$ 9%, leads to a decrease in exergy efficiency of 3%.

For an increase in the compression ration of 25%, the purchase cost of the compressor rises by 80%.

An increase in ${ɳ}_{s,cp}$ from 0.7 to 0.85 leads to an increase in the exergetic performance coefficient of 21%, while the compressor purchase cost triples.

The exergoeconomic optimization procedure, based on finding the operational and constructive solution for which the total cost formed by the sum of the operating cost and the amortization rate of the invested capital is minimal, specifies for each functional area and device the allocated exergy consumption (destruction) share.

In the paper, a direct procedure for determining the heat exchange area for each intermediate or final cooler of the compression stage is built.

Based on the NTU-ε method, graphs are built and offered. A fast and accurate way to calculate the heat exchange surfaces is provided. The procedure described in the paper is easier to use than the classic heat exchanger design method, based on the logarithmic mean temperature difference.

It is thus possible to evaluate the balance between the operating cost caused by exergy destruction and the investment cost.

## Figures and Tables

**Table 1:** Compressor input power [8] (Figure 1).

Compressor Stage	$\left|\dot{W}\right|\left[MW\right]$
1	10.292
2	10.712
3	10.751
Global fuel of the installation F	31.755

**Table 2:** Exergy destruction in the compression stages [8] (Figure 1).

Compressor Stage	$\mathrm{Exergy}\;\mathrm{Destruction}\;{\dot{I}}_{cp}\left[MW\right]$
1	1.659
2	1.647
3	1.652
$\mathrm{Total}\;{\dot{I}}_{cp}$	4.958

**Table 3:** Losses with heat exergies evacuated in the coolers of the compression stages [8] (Figure 1).

Cooler	$\left|\dot{W}\right|\left[MW\right]$
1	2.3
2	2.32
3	2.161
$\mathrm{Total}\;{\dot{L}}_{Q}$	6.781

**Table 4:** Functional characteristics of compression zone coolers (data extracted from Figure 1).

Cooler	Heat Loss	$t_{i}^{h}$	$t_{o}^{h}$	$∆T_{h}=t_{i}^{h}-t_{o}^{h}$	$∆T_{c}=t_{o}^{c}-t_{i}^{c}$	${\dot{C}}_{h}$	${\dot{C}}_{c}$
	$\dot{Q}\left[MW\right]$	[°C]	[°C]	$\left[K\right]$	$\left[K\right]$	$\left[\frac{MW}{K}\right]$	$\left[\frac{MW}{K}\right]$
1	8.35	103.4	40	63.4	9	0.1317	0.927
2	10.76	121.5	40	81.5	11.6	0.132	0.927
3	12.16	121.8	30	91.8	13.59	0.1325	0.927

**Table 5:** Functional characteristics of the Giauque–Hampson recuperative heat exchanger [37].

$T_{i}^{h}$	$T_{i}^{c}$	$T_{o}^{c}$	${\dot{C}}_{h}$	${\dot{C}}_{c}$	$C_{r}$	NEUD	$U_{o}$
$\left[K\right]$	$\left[K\right]$	$\left[K\right]$	$\left[\frac{kW}{K}\right]$	$\left[\frac{kW}{K}\right]$	[-]	[-]	$\left[\frac{kW}{m^{2}K}\right]$
300	90	294	3.69	2.358	0.6389	0.426	0.1959

## Data Availability

Data are contained within the article.

## Nomenclature

The following abbreviations are used in this manuscript:

Abbreviations	
ASU	air separation unit
COP	coefficient of performance
NTU	number of thermal units
NEUD	number of exergy units destroyed
Latin Letters	
A	heat transfer area, m^2^
C	purchase cost, Eur; heat capacity, kW/K
Cp	compressor
${\bar{c}}_{p}$	molar heat capacity, kJ/kmol/K
$\overset{´}{E}x$	current of exergy, kW
$\bar{ex}$	molar exergy, kJ/kmol
F	exergetic fuel, kW
$\bar{h}$	molar enthalpy, kJ/kmol
$\dot{I}$	current of exergy destruction due to internal irreversibility, kW
$\bar{i}$	molar exergy destruction, kJ/kmol
L	exergetic loss, kW
p	pressure, Pa
$\bar{R}$	universal constant of gases, kJ/kmol/K
$\dot{S}$	current of entropy, kJ/kg/K/s
$\bar{s}$	molar entropy, kJ/kmol/K
t	temperature, °C
T	temperature, K
U	overall heat transfer coefficient, kW/m^2^/K
$\bar{w}$	molar mechanical work, kJ/kmol
$\dot{W}$	mechanical power, kW
Subscripts	
0	environment, in equilibrium with the environment, difference between inlet temperatures in the heat exchanger
c	cold
cp	compressor
ex	exergetic
gen	generation
h	hot
i	inlet
L	loss
m	mean
max	maximum
min	minimum
o	outlet
Q	heat
Z	functional zone
Superscript	
c	cold
cp	compressor
h	Hot
M	mechanical
T	thermal
Greek Symbol	
ΔT	temperature difference
ε	thermal efficiency of the heat exchanger
${ɳ}_{s,cp}$	compression isentropic efficiency
π	compression ratio
ψ	share of an exergetic loss or destruction in the fuel consumption
